# Supplementary material for: Poultry farmer response to disease outbreaks in smallholder farming systems in southern Vietnam
Source: eLife. 2020 Aug 25;9:e59212. doi: 10.7554/eLife.59212 (PMC7505654; doi:10.7554/eLife.59212)
Supplement: Supplementary file 1. [file elife-59212-supp1.docx]

**Supplementary table 1. Fitted parameters of the original broiler chicken harvest model**

| Model | Variable | | Odds-ratio  (with 95% CI) | p-value |
| --- | --- | --- | --- | --- |
| Harvest | ONS chickens* | Same month | 1.39 (0.95 ; 2.04) | 0.09 |
|  |  | -1 month | 1.53 (1.03 ; 2.27) | 0.04 |
|  |  | -2 months | 0.69 (0.43 ; 1.09) | 0.11 |
|  | Interaction term ONS chickens* – flock size (logarithm) | Same month | 0.54 (0.37 ; 0.79) | $<{10}^{-2}$ |
|  |  | -1 month | 0.75 (0.51 ; 1.1) | 0.14 |
|  |  | -2 months | 1.57 (1.03 ; 2.41) | 0.04 |
|  | OS chickens** | Same month | 3.73 (1.36 ; 10.26) | 0.02 |
|  |  | -1 month | 1.95 (0.58 ; 6.49) | 0.28 |
|  |  | -2 months | 1.49 (0.42 ; 5.24) | 0.54 |
|  | Interaction term ONS chickens** – flock size (logarithm) | Same month | 0.37 (0.14 ; 0.99) | 0.05 |
|  |  | -1 month | 0.97 (0.25 ; 3.74) | 0.97 |
|  |  | -2 months | 1.11 (0.28 ; 4.36) | 0.89 |
|  | Number of broiler chickens in the farm (square root) | | 1.07 (1.03 ; 1.11) | $<{10}^{-2}$ |
|  | combined effect of the difference between current age and age at maturity ($\delta t$), age at maturity ($t^{*}$) and logarithm of flock size ($n$) (spline transform) | |  | $<{10}^{-3}$ |

Variables with p value <0.1 are highlighted in gray

*ONS: Outbreak with no sudden deaths

**OS: Outbreak with sudden deaths
