# Supplementary material for: Poultry farmer response to disease outbreaks in smallholder farming systems in southern Vietnam
Source: eLife. 2020 Aug 25;9:e59212. doi: 10.7554/eLife.59212 (PMC7505654; doi:10.7554/eLife.59212)
Supplement: Supplementary file 2. [file elife-59212-supp2.docx]

**Supplementary table 2. Fitted parameters of the broiler chicken harvest model with aggregated effects of outbreaks with and without sudden deaths**

| Variable |  |  | Odds-ratio  (with 95% CI) | p-value |
| --- | --- | --- | --- | --- |
| Flock  size ≤ 16 chickens | Outbreak chickens | Same month | 2.34 (1.43 ; 3.81) | $<{10}^{-3}$ |
|  |  | -1 month | 1.96 (1.14 ; 3.37) | 0.02 |
|  |  | -2 months | 0.45 (0.22 ; 0.92) | 0.03 |
|  | Number of broiler chickens in the farm (square root) | | 1.05 (1 ; 1.11) | 0.07 |
|  | combined effect of the difference between current age and age at maturity ($\delta t$) and the age at maturity ($t^{*}$) (spline transformation) | | **Supplement figure 2** | $<{10}^{-3}$ |
| Flock  size > 16 chickens | Outbreak chickens | Same month | 0.89 (0.54 ; 1.49) | 0.67 |
|  |  | -1 month | 1.15 (0.7 ; 1.88) | 0.58 |
|  |  | -2 months | 1.12 (0.7 ; 1.79) | 0.635 |
|  | Number of broiler chickens in the farm (square root) | | 1.05 (1 ; 1.11) | 0.05 |
|  | combined effect of the difference between current age and age at maturity ($\delta t$) and the age at maturity ($t^{*}$) (spline transformation) | | **Supplement figure 2** | $<{10}^{-3}$ |

Variables with p value <0.1 are highlighted in gray
