# Supplementary material for: Poultry farmer response to disease outbreaks in smallholder farming systems in southern Vietnam
Source: eLife. 2020 Aug 25;9:e59212. doi: 10.7554/eLife.59212 (PMC7505654; doi:10.7554/eLife.59212)
Supplement: Transparent reporting form [file elife-59212-transrepform.pdf]

eLIFE
1st Floor
24 Hills Road
Cambridge CB2 1JP, UK
P 01223 855340
W elifesciences.org
T @elife

eLife's transparent reporting form

We encourage authors to provide detailed information *within their submission* to facilitate the interpretation and replication of experiments. Authors can upload supporting documentation to indicate the use of appropriate reporting guidelines for health-related research (see EQUATOR Network), life science research (see the BioSharing Information Resource), or the ARRIVE guidelines for reporting work involving animal research. Where applicable, authors should refer to any relevant reporting standards documents in this form.

If you have any questions, please consult our Journal Policies and/or contact us: editorial@elifesciences.org.

Sample-size estimation

- You should state whether an appropriate sample size was computed when the study was being designed
- You should state the statistical method of sample size computation and any required assumptions
- If no explicit power analysis was used, you should describe how you decided what sample (replicate) size (number) to use

Please outline where this information can be found within the submission (e.g., sections or figure legends), or explain why this information doesn't apply to your submission:

eLife Sciences Publications, Ltd is a limited liability non-profit non-stock corporation incorporated in the State of Delaware, USA, with company number 5030732, and is registered in the UK with company number FC030576 and branch number BR015634 at the address 1st Floor, 24 Hills Road, Cambridge CB2 1JP | August 2014

There was no specific power calculation performed for the estimates obtained from study, except for the per-bird H5N1 influenza A positivity measure (prevalence) which was published as a rapid report in PLoS Currents.

This study was set up as 50 poultry farmers reporting monthly on the demographics and clinical characteristics (symptoms) of their poultry flocks, for a duration of 20 months. We knew in advance that 20 months of reporting would results in about 6 to 8 poultry cycles per farm. With 2 to 5 flocks per farm, this would lead from N=600 to N=2000 poultry flocks on which we could record data on size, duration, vaccination, hygiene, sell rate, incidence of symptoms, and frequency of outbreaks.

The frequency of disease outbreaks is irregular. The incidence of symptoms and changing patterns of sell rates were not previously known. Therefore, we were unable to choose a number of flocks (N) that would guarantee a level of statistical power under some expected conditions of future outbreak incidence and changing farmer behavior. In fact, because no previous studies had measured these characteristics, a major goal of our study was to provide the basic descriptive epidemiology of how poultry farm management changes during outbreaks periods.

The number of farms (50) was chosen together with the Ca Mau sub-Department of Livestock Production and Animal Health (CM-LPAH). This number was chosen based on study feasibility and staff availability at CM-LPAH. These types of studies are very difficult to set up in developing country settings, and Ca Mau is a rural province in southern-most part of the Mekong Delta, with no nearby research community. The number 50 was a decision made collectively by the CM-LPAH and the scientific leads on the study (OUCRU Vietnam) during several meetings held between March 2014 and March 2015.

The specific information above on the study's origins and the collaboration with CM-LPAH is not in the submission, as this is outside of the scope of the main scientific questions for the ms. We can state in the methods, if required, that the study was set up as a basic descriptive study in behavioral epidemiology and that no power calculations were done for behavioral differences in selling patterns or differences in vaccination rates as these had not been measured previously, and we had no prior expectation as to how large or small they would be.

Replicates

- You should report how often each experiment was performed
- You should include a definition of biological versus technical replication
- The data obtained should be provided and sufficient information should be provided to indicate the number of independent biological and/or technical replicates

- If you encountered any outliers, you should describe how these were handled
- Criteria for exclusion/inclusion of data should be clearly stated
- High-throughput sequence data should be uploaded before submission, with a private link for reviewers provided (these are available from both GEO and ArrayExpress)

Please outline where this information can be found within the submission (e.g., sections or figure legends), or explain why this information doesn't apply to your submission:

All data are publicly available, and all data were included in the analyses. Certain sub-analyses focused on particular poultry breeds, production types, age classes or flocks of certain sizes, but this is all clearly stated in the methods and the supplementary information file.

Data availability and link are included after the discussion section.

There were no technical or biological replicates as this was a study in behavioral epidemiology.

No sequence data in this study.

Statistical reporting

- Statistical analysis methods should be described and justified
- Raw data should be presented in figures whenever informative to do so (typically when N per group is less than 10)
- For each experiment, you should identify the statistical tests used, exact values of N, definitions of center, methods of multiple test correction, and dispersion and precision measures (e.g., mean, median, SD, SEM, confidence intervals; and, for the major substantive results, a measure of effect size (e.g., Pearson's r, Cohen's d)
- Report exact p-values wherever possible alongside the summary statistics and 95% confidence intervals. These should be reported for all key questions and not only when the p-value is less than 0.05.

Please outline where this information can be found within the submission (e.g., sections or figure legends), or explain why this information doesn't apply to your submission:

Yes, this is all described in detail in the Methods Section and Table 2. The main focus of this paper is the statistical approach to detecting an early sale under certain outbreak conditions, and we have spent considerable word count on the details and nuances of this inference.

(For large datasets, or papers with a very large number of statistical tests, you may upload a single table file with tests, Ns, etc., with reference to sections in the manuscript.)

Group allocation

- Indicate how samples were allocated into experimental groups (in the case of clinical studies, please specify allocation to treatment method); if randomization was used, please also state if restricted randomization was applied
- Indicate if masking was used during group allocation, data collection and/or data analysis

Please outline where this information can be found within the submission (e.g., sections or figure legends), or explain why this information doesn't apply to your submission:

Not applicable to our study.

Additional data files ("source data")

- We encourage you to upload relevant additional data files, such as numerical data that are represented as a graph in a figure, or as a summary table
- Where provided, these should be in the most useful format, and they can be uploaded as "Source data" files linked to a main figure or table
- Include model definition files including the full list of parameters used
- Include code used for data analysis (e.g., R, MatLab)
- Avoid stating that data files are "available upon request"

Please indicate the figures or tables for which source data files have been provided:

All data are already publicly available at https://osf.io/ws3vu/

eLife Sciences Publications, Ltd is a limited liability non-profit non-stock corporation incorporated in the State of Delaware, USA, with company number 5030732, and is registered in the UK with company number FC030576 and branch number BR015634 at the address 1st Floor, 24 Hills Road, Cambridge CB2 1JP | August 2014
